# Synthesis of monodisperse magnetic restricted microspheres for recognition of thiamphenicol in milk†

**DOI:** 10.1039/d0ra10268g

**Published:** 2021-02-10

**Authors:** Shuai Zhang, Huachun Liu, Tianpei Cai, Yanqiang Zhou, Jianmin Li, Xiaoxiao Wang, Shanwen Zhao, Chunmiao Bo, Bolin Gong

**Affiliations:** School of Chemistry and Chemical Engineering, North Minzu University No. 204 Wenchang North Street, Xixia District Yinchuan 750021 China gongbolin@163.com +86-0951-2067917 +86-0951-2067917

## Abstract

Taking thiamphenicol as the research object, a new type of magnetic restricted access molecularly imprinted polymer (RAM-MMIP) with specific recognition was prepared by a one-step swelling method. The polymer microspheres were characterized and analyzed by scanning electron microscopy, X-ray diffraction, elemental analysis, contact angle measurement and vibrating sample magnetometry. When the ratio of template molecule, functional monomer and cross linking agent was 1 : 4 : 8, the adsorption capacity reached the maximum. Under these conditions, RAM-MIP magnetic solid phase extraction (M-SPE) was combined with HPLC to analyze thiamphenicol in milk samples. Satisfactory linear correlation (*R*^2^ > 0.9977), good detection limit (LOD: 10.4 μg L^−1^), high recovery rate (96.5–101.1%), and relative standard deviation (RSD: 2.8–3.8%) were obtained. Therefore, our synthesized material can be used for the analysis of TAP in complex milk samples, and has broad application value.

## Abstract 2

Taking thiamphenicol as the research object, a new type of magnetic restricted access molecularly imprinted polymer (RAM-MMIP) with specific recognition was prepared by a one-step swelling method.

## Graphical abstract

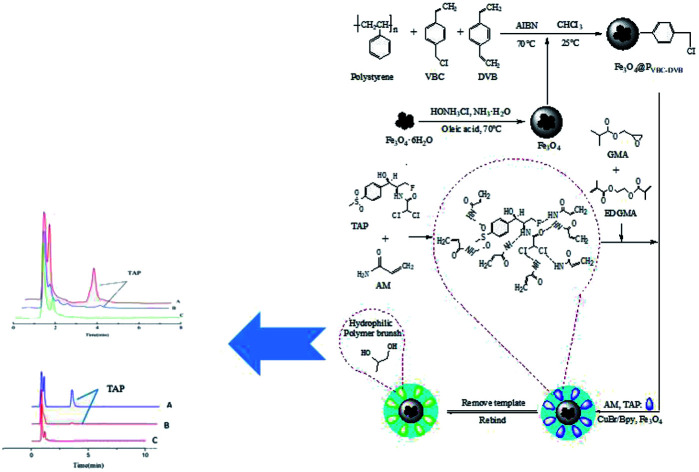

## 1. Introduction

Thiamphenicol (TAP) is a commonly used antibacterial drug with a similar structure and pharmacology to that of chloramphenicol.^1^ TAP is used for animal breeding and agricultural planting, for the purpose of inhibiting bacteria or other microorganisms, and TAP can enter the environment through feces and soil.^2^ Because TAP inhibits the production of platelets and adversely affects the blood system,^3^ it poses a huge threat to the environment and human health.^4^ As we all know, milk and related products are derived from dairy cows and dairy cows need to obtain food from the environment. These drugs will remain and accumulate in the dairy cow's body and in the environment, enriching toxicity through the food chain, and ultimately endangering human health.^5,6^ In order to protect the physical and mental health of consumers, many countries have begun to establish maximum residue limits for antibiotics.^7^ According to the Ministry of Agriculture of China, the maximum residue of TAP is 50 mg g^−1^. Therefore, it is urgent to find an effective method to quickly and effectively enrich and detect TAP in complex samples. However, considering the minimum residue limit of antibiotics in milk samples, it is very important to establish sensitive and specific analytical methods.

The main detection methods for TAP include liquid chromatography,^8^ gas chromatography,^9^ gas chromatography-tandem mass spectrometry,^10^ and liquid chromatography mass spectrometry.^11^ High performance liquid chromatography is a more suitable analytical method for the detection and separation of TAP in milk. Bitas and Samanidou^12^ used the extraction of molecularly imprinted polymers (MIPs) combined with chromatographic analysis for the detection of thiamphenicol in milk. Mohsenzadeh *et al.*^13^ reviewed the research on MIPs in antibiotics in milk. In order to reduce the cost of TAP detection and other interference, MIPs have become the object of this research.

Molecular imprinting is a preparation method for obtaining molecules that match the shape, size, and functional group of a specific target molecule in space, based on a “lock-key” principle.^14,15^ MIPs are polymer materials with special molecular recognition ability and high selectivity for certain molecules.^16^ They are formed according to the shape, size, and functional groups of the template molecule. Owing to the good chemical properties and thermal stability of MIPs,^17^ they are used in chemical analysis in different forms: amorphous,^18^ spherical particles,^19–22^ dendrimers,^23^ or polymer layers (such as magnetite or silica) coated on another medium. The technology for preparing MIPs using template molecules is very mature. When the template molecules are removed, holes in the three-dimensional structure similar to the template molecule remain in the material. The resulting imprinted polymer is more rigid and stable than the non-imprinted polymer, has a larger pH range and greater temperature flexibility, and can be used with a wider range of solvents. As the synthesis of MIPs is also relatively easy and cheap, they are a preferred substitute for natural receptors and have been widely used in the fields of chromatography, solid phase extraction, chemical sensors, *etc.*^24,25^ However, with free radical polymerization methods, the rate of chain growth cannot be controlled, and side reactions during chain transfer and chain termination cause substantial variation in the size of the polymer,^26^ making it difficult to prepare conventional MIPs. Living/controlled free radical polymerization methods such as surface-initiated atom transfer radical polymerization (SI-ATRP) can produce more uniform polymers by controlling the rate of chain growth. The magnetic MIPs (MMIPs) prepared by Li's^27^ group using SI-ATRP technology showed good selectivity for the detection of cephalosporin in water and milk. However, there are few reports about the MMIPs of monodisperse magnetic microspheres (Fe_3_O_4_@P_VBC–DVB_) prepared by SI-ATRP technology.

## 2. Experimental

### 2.1 Instruments and chemical reagents

Scanning electron microscopy (SEM; JSM-7500F, JEM Corporation, Japan) was used to observe the morphology of the polymers, and high-performance liquid chromatography (HPLC; LC-20AT, Shimadzu Corporation, Japan) was used to detect the separation of the prepared materials. An ultrasonic cell disruption instrument (DE JY-92, Ningbo Xinzhi Biological Technology Co., Ltd.) was used to completely emulsify the solutions. Concentrations of the samples were detected by ultraviolet (UV)-visible spectrophotometry (TU-1810, Beijing General Analysis General Instrument Co., Ltd.); changes in C, H, O, and N contents were measured by elemental analysis (Vario EL III, German Elementary Company); and contact angle measurements (OCA-20, German Elementary Company) were used to test the hydrophilicity of the synthetic materials.

The reagents used for the preparation of microspheres were styrene (St, 99%), 4-vinylbenzyl chloride (VBC, 90%), divinylbenzene (DVB, 80%), 2,2-azobisisobutyronitrile (AIBN, 99%), and standard analytical solutions. Florfenicol (FFC, 98%), chloramphenicol (CAP, 98%), thiamphenicol (TAP, 99%), and other reagents were supplied by Aladdin Reagent Shanghai Co., Ltd. Polyvinyl alcohol (PVA, 98.0–98.8%), hydrochloric acid, FeCl_3_·6H_2_O, FeCl_2_·4H_2_O, sodium dodecyl sulfate (SDS, >99.5%), concentrated ammonia, hydroxylamine, and tetraethyl orthosilicate were purchased from Shanghai Maclean Biochemical Co., Ltd. Polyvinylpyrrolidone (PVP, 98%) was supplied by Sigma.

### 2.2 Synthesis of St seeds

Eleven millilitres of treated St, 0.25 g AIBN and 1.5 g PVP were accurately measured and dissolved in absolute ethanol. The above solution was added to a 50 mL beaker, ultrasonically dispersed until it was uniform, and then transferred to a rotary evaporator. Nitrogen protection was used to avoid interference of oxygen in the radical reaction. After completion of the reaction, the solution was centrifuged at high speed, the supernatant was removed, the precipitate was washed with ethanol, and the product was stored in 0.2% SDS.

### 2.3 Preparation of P_VBC–DVB_ microspheres

Appropriate amounts of VBC, cross-linker DVB, initiator AIBN, and pore formers toluene and dibutyl phthalate were added to a beaker, ultrasonicated until completely dissolved, and mixed well. After addition of 0.2% SDS and 5% PVA, the mixture was sonicated again until completely dissolved. After dropwise addition of 5 mL ultra-pure water, all the solutions were mixed and emulsified with a cell disrupter until completely dissolved and no oily liquid remained. The polystyrene seed liquid was accurately measured, and the activated seed liquid was stirred at room temperature. The emulsified solution was mixed with polystyrene seed microspheres, stirred and swelled for 12 h at 30 °C, and flushed with nitrogen for 30 min. The flask was sealed and protected with nitrogen. The temperature was raised to 70 °C for 24 h. After the reaction, the reaction product was washed with distilled water at 70 °C and ethanol several times, then placed in an oven at 65 °C, followed by Soxhlet extraction for 48 h to obtain P_VBC–DVB_ microspheres.

### 2.4 Synthetic magnetic Fe_3_O_4_

The principle of magnet preparation was as follows:

1 4Fe^3+^ + 2HONH_3_ + 5H_2_O ⇌ 4Fe^2+^ + N_2_O↑ + 6H_3_O^+^

2 2Fe^3+^ + Fe^2+^ + NH_4_OH → Fe_3_O_4_ + NH_4_^+^ + H_2_O

In a beaker, 300 mL of 0.05 mol L^−1^ FeCl_3_·6H_2_O ethanol/water solution (v/v, 1 : 1) was heated to 50 °C for 15 min. Hydrochloric acid was added to the solution, with continued stirring of the reaction mixture for 10 min; then, ammonia water was dripped into the solution until the pH was greater than 9–10. The pH adjustment was followed by stirring for a further 30 min, after which 5 mL of oleic acid was added dropwise and the stirring was continued for 10 min. The temperature was raised to 70 °C, and the solution was heated for 30 min before being cooled to room temperature and allowed to stand. Magnetic field separation was performed with an external magnetic field, and the solution was washed with anhydrous ethanol several times until transparent. A black precipitate was obtained and dried under vacuum at 65 °C to obtain magnetic Fe_3_O_4_.

### 2.5 Synthesis of Fe_3_O_4_@P_VBC–DVB_

Ten grams of P_VBC–DVB_ microspheres were mixed with chloroform and swelled at room temperature for 24 h before the addition of 3.0 g of Fe_3_O_4_ microspheres. The mixed solution was dispersed by ultrasound for 90 min and mechanically stirred for 4 h. After the reaction, magnetic separation was used to obtain magnetic microspheres, which were dried under vacuum at 65 °C.

### 2.6 Preparation of TAP–RAM-MMIPs

2 g of Fe_3_O_4_@P_VBC–DVB_, 0.07 g CuBr and 0.21 g 2,2-bipyridine were sealed in a round-bottomed flask containing 40 mL of water/acetonitrile (v/v, 1 : 4) as the reaction solvent, 1.12 mol of template molecule TAP, 4 mmol of functional monomer acryl amide, and 15 mmol of cross-linker ethylene glycol alcohol dimethacrylate. The prepared solution was pre-polymerized for 5 h at room temperature. Then, 2.0 mmol GMA and 0.46 mmol AIBN were added, and the solution was uniformly dispersed by ultrasound. Nitrogen was introduced into the reaction solution for 30 min, after which it was warmed to 60 °C for 24 h. After the reaction was completed, the obtained product was sequentially washed three times with methanol and 0.1 mmol L^−1^ disodium ethylenediamine tetra acetate, and then dried under vacuum at 65 °C. Then, 1.0 g of the product was mixed with 0.1 mol L^−1^ sulfuric acid solution to perform a ring-opening reaction at 60 °C for 12 h. After completion of the reaction, a magnetic field was applied for separation, followed by washing with distilled water and ethanol three times, and vacuum drying at 65 °C. The ring-opened product was subjected to Soxhlet extraction by washing with a methanol/glacial acetic acid (v/v, 4 : 1) solution for 48 h. After Soxhlet extraction, the product was washed three times with methanol and vacuum-dried at 65 °C to obtain TAP–RAM-MMIPs. The TAP–RAM-MNIPs were prepared as described above, except that no TAP template was added.

### 2.7 Adsorption experiments

#### 2.7.1 Isothermal adsorption

Eight samples of 0.02 g TAP–RAM-MMIPs and TAP–RAM-MNIPs were weighed separately, and methanol was used as a solvent to prepare TAP solutions of different concentrations. Samples were mixed with the TAP solutions, sealed in 50 mL Erlenmeyer flasks, and shaken for 24 h at constant room temperature. After the oscillating adsorption was completed, magnetic field separation was performed; the supernatant was filtered through a 0.45 μm filter, the liquid was diluted to 0.6 mg mL^−1^, and the absorbance was measured with a UV spectrophotometer. The average adsorption amount (*Q*, mg g^−1^) of the polymer was calculated by the following formula:2-1
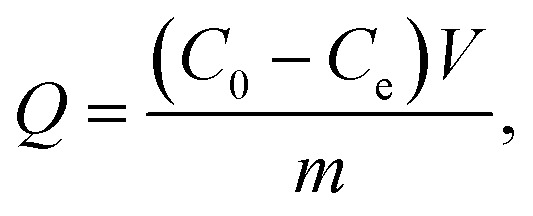
where *C*_0_ is the initial concentration (mg L^−1^), *C*_e_ is the concentration at the adsorption equilibrium (mg L^−1^), *V* is the volume of the solution (L), and *m* is the mass of the polymer (g).

#### 2.7.2 Kinetic adsorption study

Eight 0.02 g samples of TAP–RAM-MMIPs and TAP–RAM-MNIPs were weighed, and a 150 mL L^−1^ TAP solution was prepared. Samples were mixed with the TAP solution, sealed in 50 mL Erlenmeyer flasks, and shaken in a thermostatic shaker. Each group of adsorption capacity test for 10 minutes, magnetic field separation was performed, and the supernatant was filtered through a 0.45 μm filter. The liquid was diluted to 0.6 mg mL^−1^, the absorbance was measured with a UV spectrophotometer, and the adsorption of the polymer at different time points was calculated by formula (2-1).

#### 2.7.3 Competitive selective adsorption study

Three 0.02 g samples of TAP–RAM-MMIPs and TAP–RAM-MNIPs were weighed, and 150 mL L^−1^ solutions of TAP, CAP, and FF were prepared. Samples were mixed with these solutions, sealed in 50 mL Erlenmeyer flasks and shaken for 12 h in a thermostatic shaker. Magnetic separation was performed for each group, and the supernatant was filtered with a 0.45 μm filter. The filtered liquid was diluted to a certain concentration, the absorbance was measured with a UV spectrophotometer, and formula (2-1) was used to calculate the adsorption amounts of the polymer.

### 2.8 Processing of samples

Milk and river water without pre-treatment and standard TAP were used as blank samples. 5 mL milk sample and river water were accurately measured and transferred to a 50 mL centrifuge tube; methanol-glacial acetic acid (7/3, v/v) mixture was added, vortexed for 2 minutes, and ultrasonically extracted for 10 minutes. The supernatant was filtered with a membrane filter and stored in a centrifuge tube for later use. Different quantities of the activated TAP–RAM-MMIPs and TAP–RAM-MNIPs were added to the spiked milk and river water for 30 min. Different ratios of methanol and acetic acid were used as eluent, and the eluent was collected and dried, reconstituted with the mobile phase, and analyzed by HPLC.

### 2.9 HPLC chromatography conditions

A C_18_ column (150 mm × 4.6 mm, 5 μm; Shimadzu Company, Japan) was used with borax/methanol (v/v, 2 : 3) as the mobile phase. The borax solution (10.0 mmol L^−1^) was adjusted with 85% phosphoric acid solution (pH = 6.0). The HPLC parameters were as follows: detection wavelength, 225 nm; column temperature, 25 °C; flow rate, 1.0 mL min^−1^; injection volume, 20 μL.

## 3. Results and discussion

### 3.1 Synthetic TAP–RAM-MMIPs

First, polystyrene microspheres were used as seeds to carry out swelling polymerization in aqueous solution. Single-dispersion P_VBC–DVB_ was prepared by a one-step seed swelling method, and magnetic Fe_3_O_4_ nanocomposites coated with oleic acid were synthesized by co-precipitation. P_VBC–DVB_ microspheres were swelled with chloroform, and Fe_3_O_4_ particles were used to fill the P_VBC–DVB_ microspheres. CuBr/Bpy was used to form the experimental catalytic system. TAP–RAM-MMIPs were prepared in a mixed acetonitrile/water solution.

**Fig. 1:**
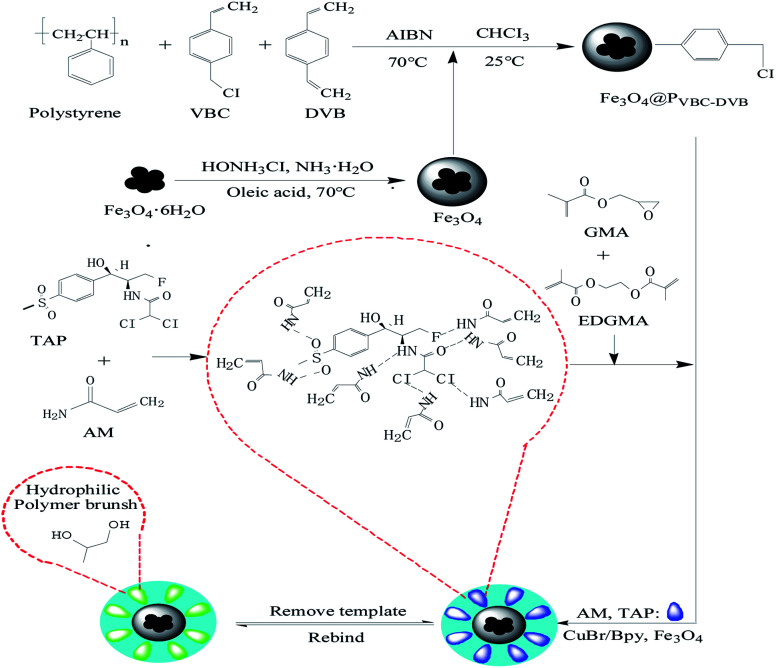
Synthetic route for the preparation of RAM-MIPs.

Using magnetic microspheres (Fe_3_O_4_@P_VBC–DVB_) as carriers, SI-ATRP technology was used to initiate free radical polymerization in the reaction solution, and the pre-polymers formed by template molecules, functional monomers, and cross-linking agents^28^ were grafted onto the surface of Fe_3_O_4_@P_VBC–DVB_ microspheres. Template molecules were removed to obtain RAM-MMIPs. The synthetic route is shown in Fig. 1.

### 3.2 Characterization and analysis of RAM-MMIPs

#### 3.2.1 Scanning electron microscopy characterization and analysis

**Fig. 2:**
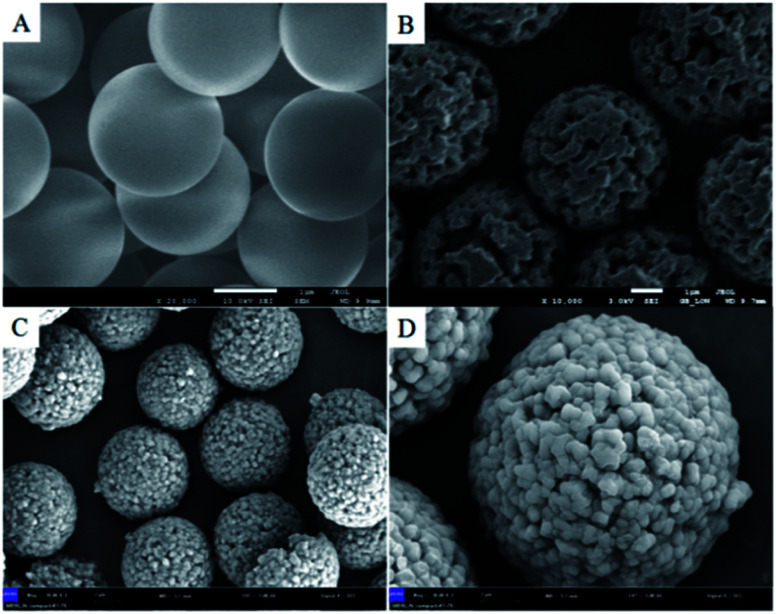
(A) Seed microspheres, (B) P_VBC–DVB_ microspheres and (C and D) TAP–RAM-MMIPs microspheres.

According to SEM analysis, Fe_3_O_4_@P_VBC–DVB_ microspheres and TAP–RAM-MMIP polymer microspheres both had good mono-dispersity and uniform size. Fig. 2A shows the seed microspheres, which had relatively smooth surfaces and particle size of about 2.1 μm. The preparation of seed microspheres met the experimental requirements. Fig. 2B shows the P_VBC–DVB_ microspheres, which had a rough surface and multiple apertures; the particle size was about 4.2 μm, substantially larger than that of the seed microspheres, indicating that the P_VBC–DVB_ microspheres had been successfully prepared. Fig. 2C and D show TAP–RAM-MMIPs, which had similar properties as the microspheres. Compared with Fig. 2B, the surface of the microspheres showed some changes owing to the different functional groups grafted inside and outside the microspheres, making them more diverse in nature and function. As shown in Fig. 2D, the microspheres were porous, but their pore size was significantly smaller than that of the microspheres shown in Fig. 2B. As the microspheres contained different substances, the pore size became smaller in order to “lock” the substance inside. As shown in Fig. 2D, the polymer microspheres were larger than any of the other microspheres. Thus, the preparation of polymer microspheres successfully produced the morphology required for the experiments.

#### 3.2.2 Hydrophilic analysis study

**Fig. 3:**
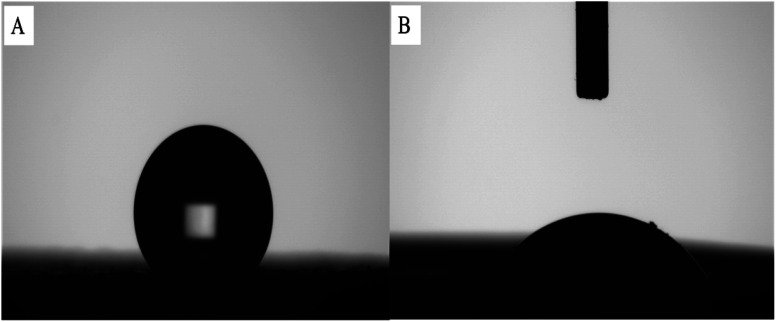
(A) P_VBC–DVB_ microspheres and (B) TAP–RAM-MMIP microspheres.

Contact angle measurements were performed on P_VBC–DVB_ microspheres and TAP–RAM-MMIP microspheres. Fig. 3A shows the contact angle of the P_VBC–DVB_ microspheres. As both the monomer and the cross-linking agent of the synthetic microspheres were hydrophobic reagents, the synthesized microspheres were hydrophobic (contact angle > 90°). Fig. 3B shows the contact angle of the TAP–RAM-MMIP microspheres. The synthetic polymer had a hydroxyl layer combined with the polymer's open pores, making it a hydrophilic material (contact angle < 90°). These results prove that the microspheres and polymers were successfully prepared.

#### 3.2.3 Analysis of magnetic properties of TAP–RAM-MMIPs

The polymer was dissolved in absolute ethanol. It can be clearly seen from Fig. S1A† that the solution in the beaker was turbid. After placing the magnet on one side for a few minutes, as shown in Fig. S1B,† the beaker solution became clear and the polymer was completely adsorbed on the beaker wall. This indicates that the polymer had good magnetic properties.^29^

#### 3.2.4 Elemental analysis

The changes in the amounts of elements H, C, and N contained in the P_VBC–DVB_ microspheres and the TAP–RAM-MMIPs polymer are shown in Table 1. The increases in H, C, and N indicate that the monomer and cross-linking agent were successfully grafted onto the polymer surface. The grafting amount (35.29 μmol m^−1^) was calculated using formula (2-2); the specific surface area of the imprinted polymer was 491.93 m^2^ g^−1^.2-2
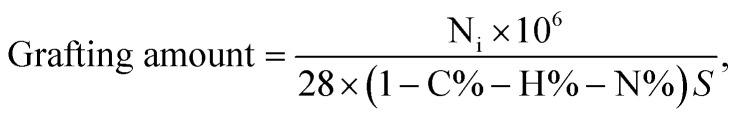
where N_i_ is the percentage increase of the element, N% is the percentage increase after grafting, and *S* is the specific surface area.

**Table 1:** Elemental analysis of P_VBC–DVB_ microspheres and TAP–RAM-MMIPs polymers

Material	Elemental composition (%, w/w)		
	C	N	H
P_VBC–DVB_	71.39	0.74	5.38
TAP–RAM-MMIPs	71.84	0.76	5.55

#### 3.2.5 X-ray diffraction analysis and characterization

The results of the XRD analysis of Fe_3_O_4_ and MMIPs are shown in Fig. S2A and B,† respectively. In the diffraction analysis of Fe_3_O_4_, six characteristic peaks were observed at 2*θ* = 30.08, 35.56, 43.18, 53.82, 57.12, and 62.88. The corresponding diffraction lines were determined using a standard X-ray diffraction analysis chart. The faces corresponded to 220, 311, 400, 422, 511, and 400, respectively, indicating that Fe_3_O_4_ had anti-spinal structural properties. The MMIP showed very similar characteristic peaks, with a slight reduction in the peak intensity, indicating that the MMIP had anti-stiff structural properties. It is proved that our monodisperse microspheres not only contain Fe_3_O_4_, but also graft other different groups. The structure and properties of anti spinel are not changed, but the absorption peak intensity of the X-ray diffraction peak is changed.

### 3.3 Selection of cross-linker dosages

Different proportions of the cross-linking agent solutions were prepared for the adsorption experiments, as shown in Fig. S3.† The contents of functional monomers and template molecules were left unchanged. The best adsorption performance was achieved when the ratio of template molecule, functional monomer, and cross-linker was 1 : 4 : 8. The smallest ratio was associated with the poorest adsorption performance of the polymer on TAP. This indicates that when the content of the cross-linking agent is low, the polymer may obtain fewer binding sites, resulting in poor adsorption performance. However, an excessive increase in proportion will also lead to a decrease in the adsorption performance. As increasing the cross-linking agent content led to an increase in the non-specific adsorption capacity, which reduced the specific adsorption capacity of the polymer for the template molecule, we chose a ratio of 1 : 4 : 8 for the experiments.

**Fig. 4:**
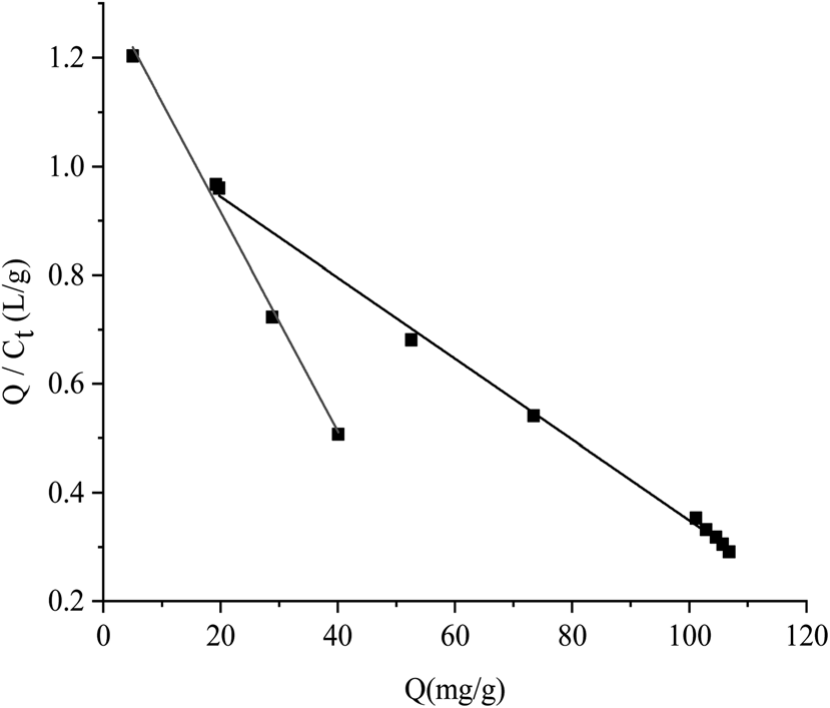
Scatchard fitting curve for MMIPs.

All the data shown in Fig. S3† were calculated by the Scatchard formula and are plotted as a Scatchard diagram as shown in Fig. 4, where the abscissa is *Q* and the ordinate is *Q*/*C*_*t*_. There were two different binding sites in the MMIPs, with linear fitting equations *y* = −0.007490*x* + 1.09308 (regression coefficient (*R*^2^) = 0.9977) and *y* = −0.02018*x* + 1.320 (*R*^2^ = 0.9937). Using the slope and intercept of the fitted equations, the maximum apparent adsorption capacities (*Q*_max_) were calculated to be 154.40 and 66.34 mg g^−1^, respectively, with dissociation constants (*K*_*t*_) of 144.11 mg L^−1^ and 48.99 mg L^−1^, respectively.2-3
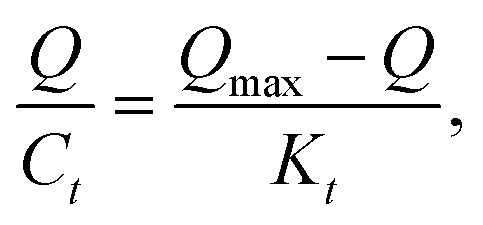
where *Q* is the adsorption equilibrium concentration (mmol L^−1^), *Q*_max_ is the maximum apparent adsorption amount (mg g^−1^), and *K*_*t*_ is the equilibrium dissociation constant (mg L^−1^).

### 3.4 Adsorption performance study

#### 3.4.1 Isothermal adsorption analysis

**Fig. 5:**
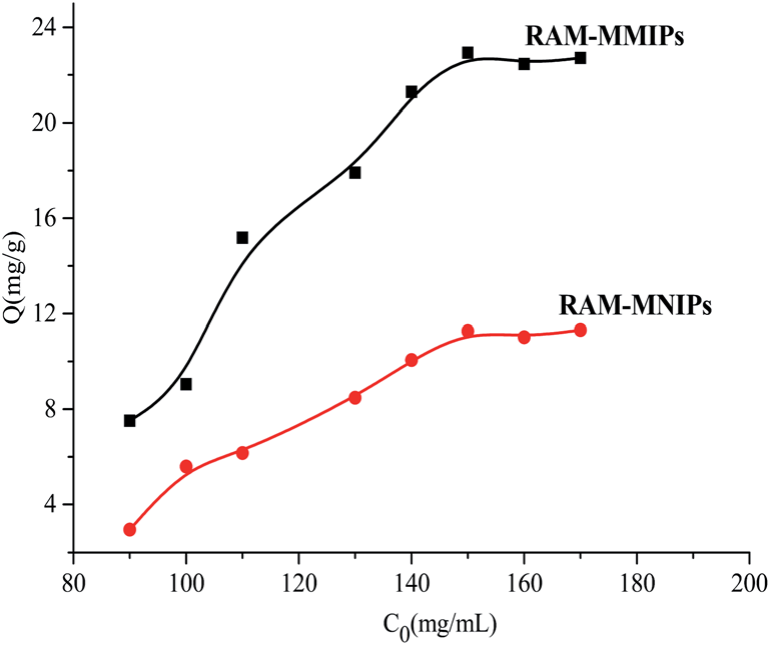
Isothermal adsorption curves for RAM-MMIPs and RAM-MNIPs.

The results of isothermal adsorption experiments on the RAM-MMIPs and RAM-MNIPs are shown in Fig. 5. For both the polymers, when the concentration reached 150 mg L^−1^, the imprinted polymer began to reach adsorption equilibrium. The maximum saturation equilibrium adsorption capacity of the RAM-MMIPs was 22.89 mg g^−1^, whereas that of the RAM-MNIPs was 11.28 mg g^−1^. The adsorption equilibrium of the imprinted polymer was also much higher than that of the non-imprinted polymer. This proves that the imprinted polymers can specifically recognize not only the template molecules but also the imprinted holes. The non-imprinted polymer had no holes and thus did not specifically recognize the template molecules. These results indicate that the non-imprinted polymers only have physical adsorption capacity, whereas the imprinted polymers also have good isothermal adsorption effects.

#### 3.4.2 Dynamic adsorption analysis

**Fig. 6:**
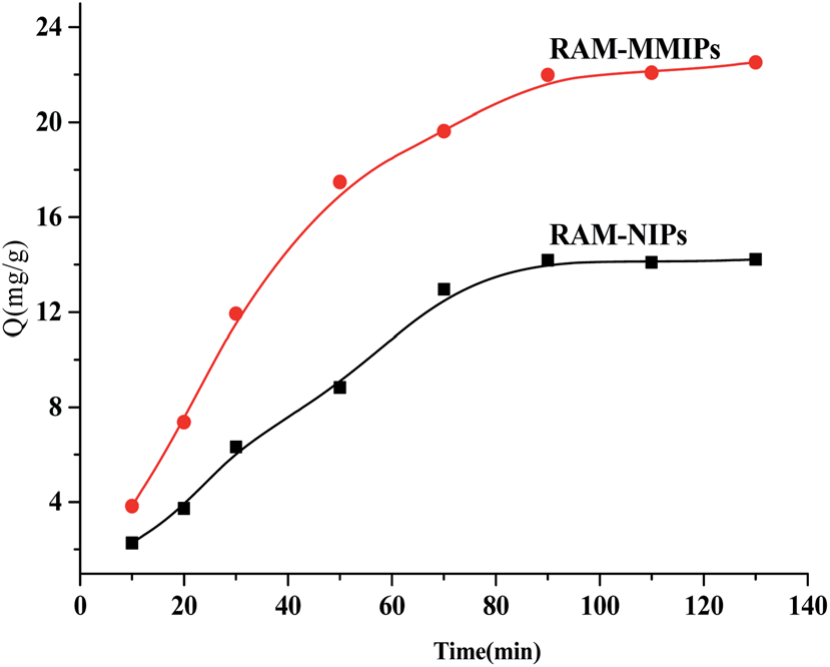
Dynamic adsorption curves for RAM-MMIPs and RAM-MNIPs.

Dynamic adsorption experiments were performed on the RAM-MMIPs and RAM-MNIPs, as shown in Fig. 6. The imprinted and non-imprinted polymers both reached saturation equilibrium in 90 min. The adsorption rate of the imprinted polymer was higher than that of the non-imprinted one, indicating that the specific property of the magnetically bound imprinted polymer was better. The results showed that the magnetic polymer had uniform size and good recognition of holes.

#### 3.4.3 Study on competitive selective adsorption analysis

Both CAP and FP were selected as the template molecules for competitive selective adsorption. Their structures are similar to that of TAP, as shown in Fig. S4.†

**Fig. 7:**
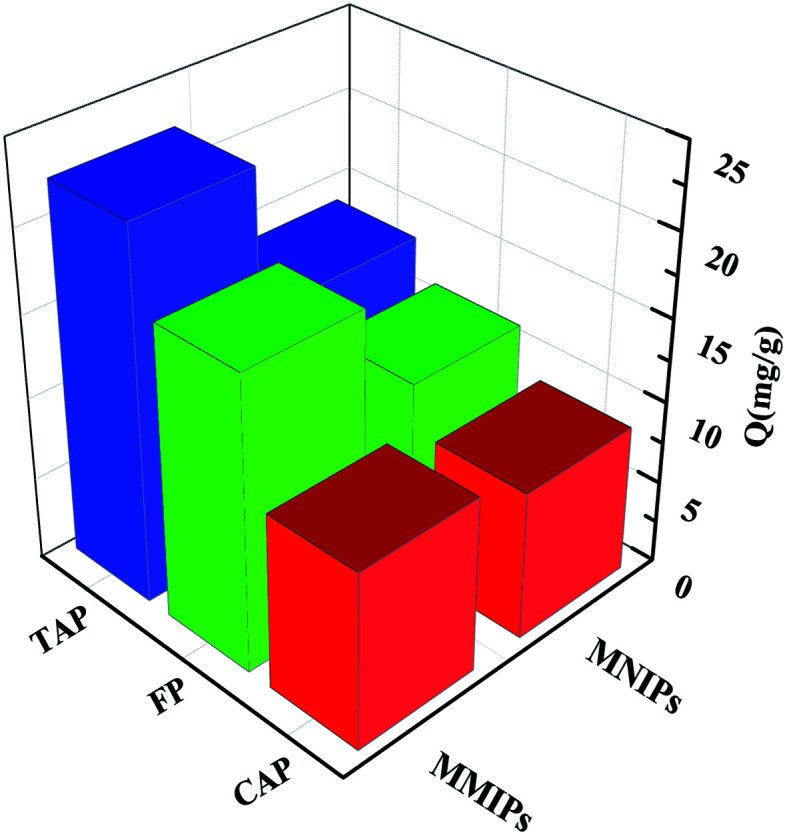
Competitive selective adsorptions.

Competitive selective adsorption experiments were performed on RAM-MMIPs and RAM-MNIPs (Fig. 7). The adsorption amounts of the three RAM-MNIPs were similar, but that of RAM-MMIPs was significantly higher than that of the corresponding RAM-MNIPs. This indicates that MIPs have higher specific recognition capabilities than magnetic non-imprinted polymers. The comparison between the three RAM-MMIPs showed that the adsorption capacity of the TAP–RAM-MMIPs was much higher than those of the other two. Therefore, the imprinted material had good selectivity, and the imprinted holes could select only the corresponding template molecules.

### 3.5 Protein exclusion analysis

**Fig. 8:**
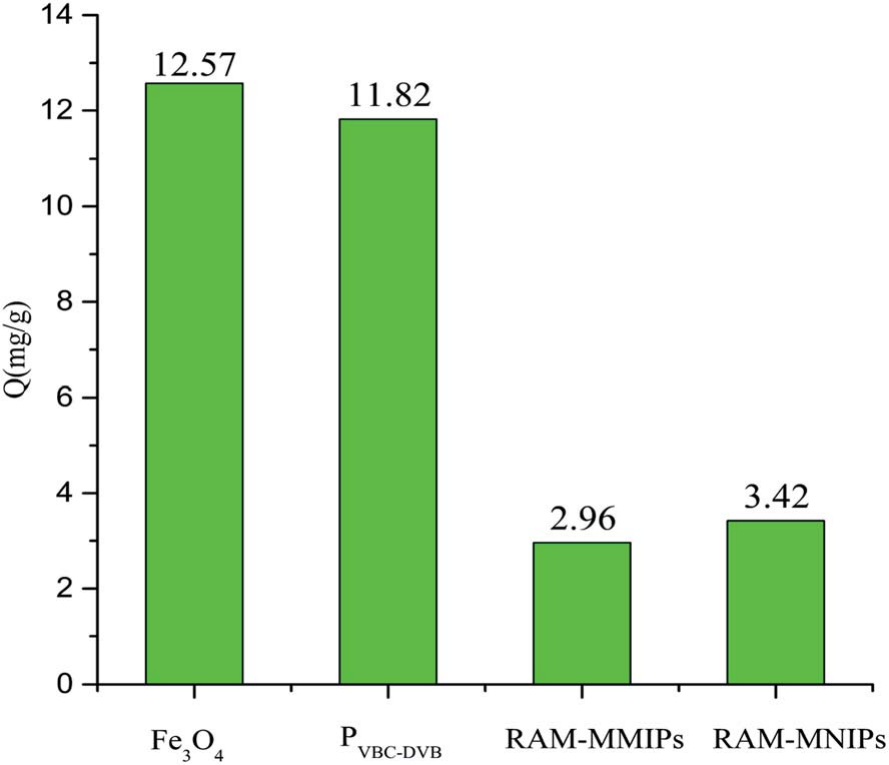
Protein exclusion adsorptions of Fe_3_O_4_, magnetic P_VBC–DVB_, RAM-MMIPs and RAM-MNIPs.

Protein exclusion experiments were performed on Fe_3_O_4_, magnetic polymer P_VBC–DVB_, RAM-MMIPs, and RAM-MNIPs, as shown in Fig. 8. The exclusion adsorption capacities of the four substances on the proteins were as follows: Fe_3_O_4_ > P_VBC–DVB_ > RAM-MNIPs > RAM-MMIPs. RAM-MNIPs and RAM-MMIPs had similar adsorption capabilities. As the RAM-MNIPs and RAM-MMIPs had limited layers, their exclusion capability was enhanced, confirming the superior exclusion capability of our restricted-imprinted polymers.

### 3.6 Optimization of SPE column parameters

The choice and ratio of the elution solvent are critical for the influence of the adsorption material. Strong acid solvents and polar solvents can easily destroy the interaction between the adsorption material and the target molecule. At the same time, different quality of adsorption materials also have a great influence on the adsorption. The experiment process is as follows: when one parameter is studied, the other parameters have fixed values.

#### 3.6.1 The quality of the adsorbent material

In order to get a better recovery rate, we put 5, 10, 15, 20, 25 and 30 mg of RAM-MIPs in 5 mL of spiked milk and river water for adsorption measurement as shown in Fig. S5 and S6.† The experimental results showed that in the milk sample, when 25 mg of RAM-MIPs were taken, the recovery rate was 92.8%. In the river water sample, when 15 mg of RAM-MIPs was taken, the recovery rate was 95.8%.

#### 3.6.2 The effect of pH

The pH value is a very important factor affecting the sample. In this study, it was optimized by adjusting the pH value of the milk and water sample solution from 4.0 to 9.0. As shown in Fig. S7 and S8,† when pH = 7, the recovery rate of milk and river water reached the maximum of 95.78% and 98.8%, respectively.

### 3.7 Application to real samples

**Fig. 9:**
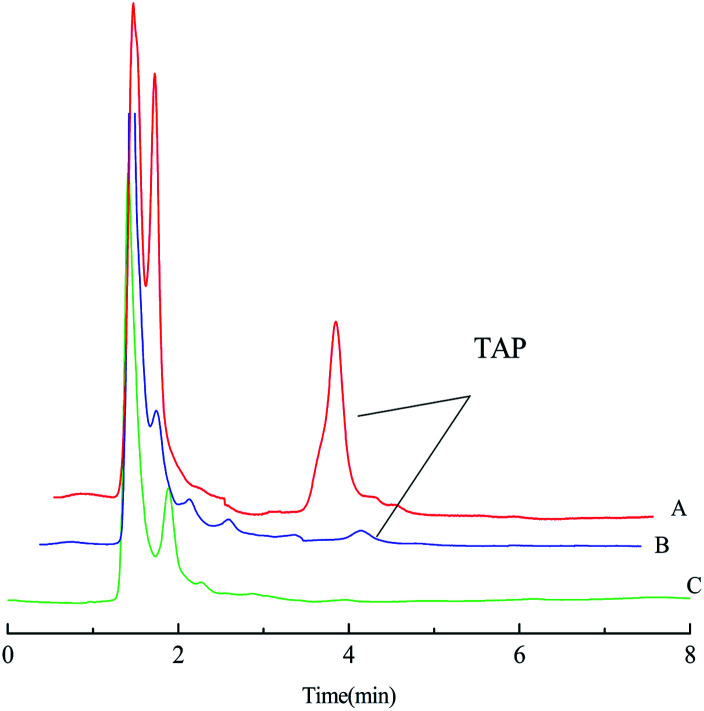
(A) Spiked milk samples after RAM-MMIP extraction. (B) Spiked milk samples after RAM-MNIP extraction and (C) blank milk sample.

**Fig. 10:**
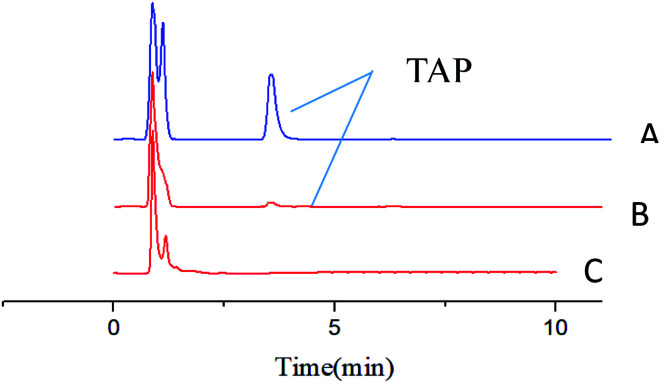
(A) Spiked river water samples after RAM-MMIP extraction. (B) Spiked river water samples after RAM-MNIP extraction and (C) blank river water sample.

The enrichment effect of TAP in milk and river water was detected by HPLC. Fig. 9A and B as well as Fig. 10A and B show the chromatograms for the RAM-MMIPs and RAM-MNIPs, corresponding to the effluent passing through the SPE cartridge. Fig. 9C shows the results for a blank sample of milk, and Fig. 10C shows a blank river water sample. It can be seen from the chromatograms that the enrichment ability of RAM-MMIPs is much higher than that of RAM-MNIPs. As the RAM-MMIPs could specifically recognize the holes of TAP, it had good enrichment and separation ability. The RAM-MNIPs were only physically adsorbed, so the absorption peaks were not obvious. The results indicate that the synthesized RAM-MMIPs possess a better separation effect.

The sample addition method was used to process the milk and river water after the addition of TAP. The results are shown in Tables 2 and 3. The recovery rate for RAM-MIPs was 96.5–101.1%, with a relative standard deviation (RSD) of 2.8–3.8%. The limit of detection (LOD) of TAP was 10.4 μg L^−1^ and 7.9 μg L^−1^ by triple signal-to-noise ratio (S/N), which is far below the detection limit of 10 mg L^−1^ reported in literature.^30^ The maximum residue level below the limit of TAP set by the Ministry of Agriculture of my country is 50 mg g^−1^. Therefore, RAM-MMIPs are suitable for use in the detection of actual samples.

**Table 2:** Spike recovery experiment for milk samples

Adsorbent	Scaling amount (μg mL^−1^)	Recovery (%)	RSD (%)
RAM-MMIPs	10	97.8	3.8
	50	96.5	3.0
	100	101.1	2.8
RAM-MNIPs	10	80.2	3.9
	50	82.5	3.6
	100	79.8	4.4

**Table 3:** Spike recovery experiment for river water samples

Adsorbent	Scaling amount (μg mL^−1^)	Recovery (%)	RSD (%)
RAM-MMIPs	10	98.0	3.6
	50	99.6	2.3
	100	103.7	3.8
RAM-MNIPs	10	86.5	2.5
	50	90.8	4.7
	100	94.6	5.0

## 4. Conclusions

In this study, we synthesized monodisperse P_VBC–DVB_ polymer microspheres and magnetic Fe_3_O_4_. The SI-ATRP technology combines the two to prepare RAM-MMIP material with specific recognition. Compared with other reported materials, this material has simple preparation conditions, fast adsorption speed, large binding capacity, lower detection limit, and high recovery rate. Studies have shown that RAM-MMIPs can be used as magnetic solid-phase extraction materials to achieve rapid and effective separation of TAP in complex samples of milk and river water by HPLC. Therefore, this material has broad application prospects for the detection and separation of TAP in milk.

## Conflicts of interest

There are no conflicts to declare.

## Supplementary Material

RA-011-D0RA10268G-s001
