# Supplementary material for: Synthesis of monodisperse magnetic restricted microspheres for recognition of thiamphenicol in milk
Source: RSC Adv. 2021 Feb 10;11(12):6869–76. doi: 10.1039/d0ra10268g (PMC8695050; doi:10.1039/d0ra10268g)
Supplement: RA-011-D0RA10268G-s001 [file RA-011-D0RA10268G-s001.pdf]

Electronic Supplementary Material (ESI) for RSC Advances.
This journal is © The Royal Society of Chemistry 2021

Synthesis of Monodisperse Magnetic Restricted Microspheres for Recognition of Thiamphenicol in Milk

Shuai Zhang, Huachun Liu, Tianpei Cai, Yanqiang Zhou, Jianmin Li, Xiaoxiao Wang, Shanwen Zhao, Chunmiao Bo and Bolin Gong*

School of Chemistry and Chemical Engineering, North Minzu University, Yinchuan 750021, China.

*Prof. Bolin Gong

No. 204 Wenchang North Street, Xixia District, Yinchuan City, China

Tel: +86-0951-2067917

Fax: +86-0951-2067917

E-mail: gongbolin@163.com

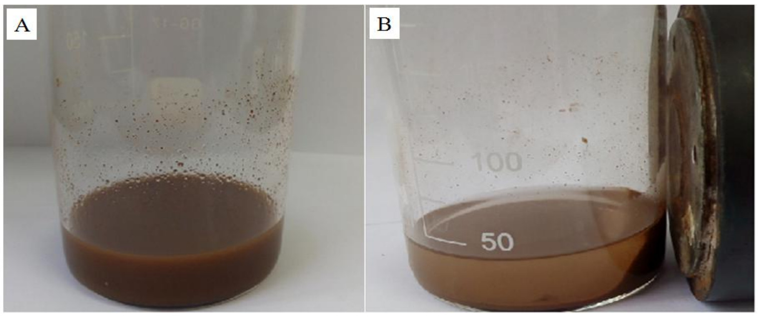

Fig. S1. Analysis of magnetic properties of polymers.

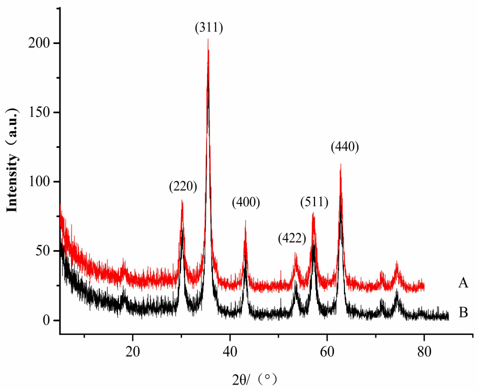

Fig.S2. X-ray diffraction analysis of (A) Fe_3O_4, (B) MMIPs.

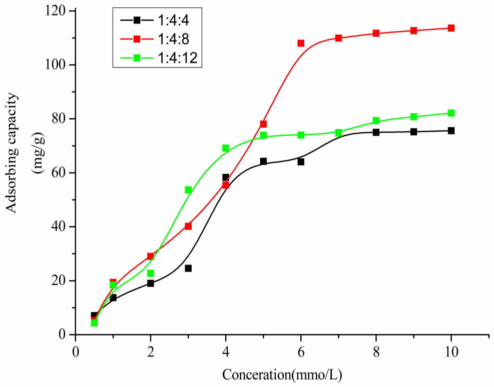

Fig. S3. Effects of different amounts of cross-linking agents on the adsorption capacity of MMIPs.

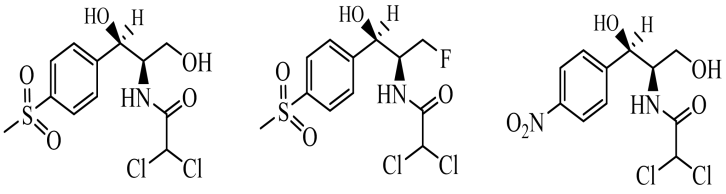

TAP **FP** **CAP**

Fig. S4. Competitive target molecular structures.

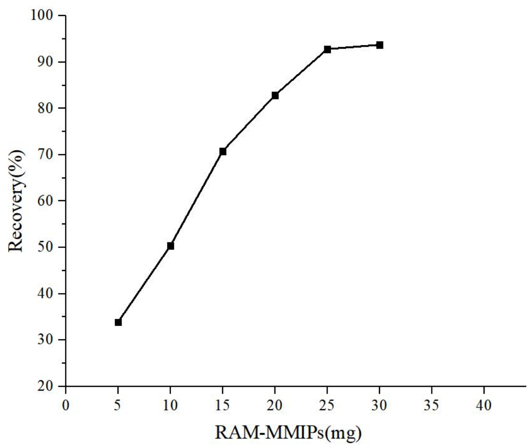

Fig. S5. The effects of the amounts of RAM-MMIPs on spiked milk

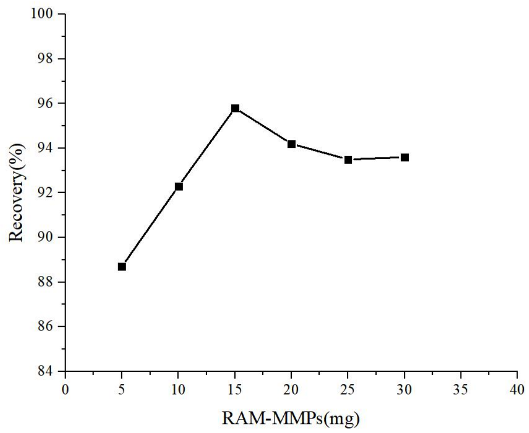

Fig. S6. The effects of the amounts of RAM-MMIPs on spiked river water

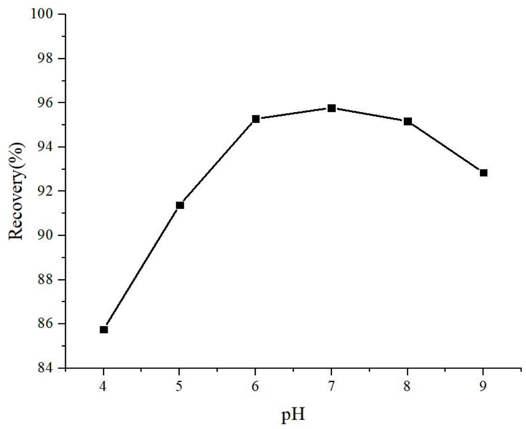

Fig. S7. The effects of pH of sample solution on spiked milk

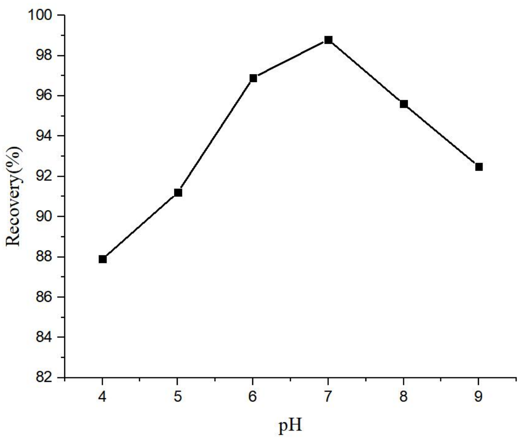

Fig. S8. The effects of pH of sample solution on spiked river water
